# Association of Pneumococcal Conjugate Vaccination With Severe Acute Respiratory Syndrome Coronavirus 2 Infection Among Older Adult Recipients of Coronavirus Disease 2019 Vaccines: A Longitudinal Cohort Study

**DOI:** 10.1093/infdis/jiae387

**Published:** 2024-08-05

**Authors:** Joseph A Lewnard, Vennis Hong, Lindsay R Grant, Bradley K Ackerson, Katia J Bruxvoort, Magdalena Pomichowski, Adriano Arguedas, Alejandro Cané, Luis Jodar, Bradford D Gessner, Sara Y Tartof

**Affiliations:** Center for Computational Biology, School of Public Health; College of Statistics, Data Science, and Society; Augmented Graduate Group in Computational Precision Health, University of California, Berkeley; Department of Research and Evaluation, Kaiser Permanente Southern California, Pasadena; Pfizer Vaccines, Collegeville, Pennsylvania; Department of Research and Evaluation, Kaiser Permanente Southern California, Pasadena; Department of Epidemiology, School of Public Health, University of Alabama at Birmingham; Department of Research and Evaluation, Kaiser Permanente Southern California, Pasadena; Pfizer Vaccines, Collegeville, Pennsylvania; Pfizer Vaccines, Collegeville, Pennsylvania; Pfizer Vaccines, Collegeville, Pennsylvania; Pfizer Vaccines, Collegeville, Pennsylvania; Department of Research and Evaluation, Kaiser Permanente Southern California, Pasadena; Department of Health Systems Science, Kaiser Permanente Bernard J. Tyson School of Medicine, Pasadena, California

**Keywords:** pneumococcal conjugate vaccine, *Streptococcus pneumoniae*, SARS-CoV-2, polymicrobial infections, adult vaccination

## Abstract

**Background:**

Pneumococcal carriage is associated with increased acquisition and duration of severe acute respiratory syndrome coronavirus 2 (SARS-CoV-2) infection among adults. While pneumococcal conjugate vaccines (PCVs) prevent carriage of vaccine-serotype pneumococci, their potential impact on coronavirus disease 2019 (COVID-19)–related outcomes remains poorly understood in populations with prevalent immunity against SARS-CoV-2.

**Methods:**

We undertook a retrospective cohort study of adults aged ≥65 years in the Kaiser Permanente Southern California healthcare system who had received ≥2 COVID-19 vaccine doses, comparing risk of SARS-CoV-2 infection between 1 January 2021 and 31 December 2022 among recipients and nonrecipients of 13-valent PCV (PCV13) employing multiple strategies to mitigate bias from differential test-seeking behavior.

**Results:**

The ajusted hazard ratio of confirmed SARS-CoV-2 infection comparing PCV13 recipients to nonrecipients was 0.92 (95% confidence interval [CI], .90–.95), corresponding to prevention of 3.9 (95% CI, 2.6–5.3) infections per 100 person-years. Following receipt of 2, 3, and ≥4 COVID-19 vaccine doses, aHRs (95% CI) were 0.85 (.81–.89), 0.94 (.90–.97), and 0.99 (.93–1.04), respectively. The aHR (95% CI) for persons who had not received COVID-19 vaccination in the preceding 6 months was 0.90 (.86–.93), versus 0.94 (.91–.98) within 6 months after COVID-19 vaccination. Similarly, aHRs (95% CI) were 0.92 (.89–.94) for persons without history of documented SARS-CoV-2 infection, versus 1.00 (.90–1.12) for persons with documented prior infection.

**Conclusions:**

Among older adults who had received ≥2 COVID-19 vaccine doses, PCV13 was associated with modest protection against SARS-CoV-2 infection. Protective effects of PCV13 were greater among individuals expected to have weaker immune protection against SARS-CoV-2 infection.

Polymicrobial infections involving viral and bacterial pathogens account for substantial disease burden among children and adults. While secondary bacterial pneumonia and invasive disease occurring after primary infection with influenza or other respiratory viruses are a well-understood pathway of viral–bacterial interactions [1], severe bacterial coinfections and secondary infections have not commonly been detected in association with severe acute respiratory syndrome coronavirus 2 (SARS-CoV-2) [2]. However, several studies undertaken during early phases of the coronavirus disease 2019 (COVID-19) pandemic identified facilitative relationships between mild or asymptomatic SARS-CoV-2 infection and colonization with *Streptococcus pneumoniae* (pneumococcus) [3, 4]. Moreover, in longitudinal cohort studies, pneumococcal carriers experienced increased rates of SARS-CoV-2 acquisition and longer durations of SARS-CoV-2 shedding in comparison to noncarriers [5], as well as dampened mucosal and cellular immune responses to SARS-CoV-2 following infection [3]. These findings resemble observed pathways of interaction between colonizing bacteria and other respiratory viral pathogens [6–8].

Given evidence from these studies that pneumococcal carriage may modulate the acquisition or clearance dynamics of SARS-CoV-2, factors modifying individuals’ risk of carrying pneumococci could be expected to impact SARS-CoV-2 infection and related clinical outcomes. We previously reported that older adults in the United States (US) who had received 13-valent pneumococcal conjugate vaccine (PCV13) experienced lower rates of COVID-19 diagnoses during the spring and summer of 2020 in comparison to adults who had not received PCV13 [9]. This finding is consistent with evidence that PCV13 reduces individuals’ risk of acute respiratory infections with numerous viral pathogens among both children and adults [10–12], including alphacoronaviruses and betacoronaviruses [13, 14]. However, it is unclear whether clinically relevant effects of PCV13 would be expected in populations with access to COVID-19 vaccines, which reduce recipients’ risk of SARS-CoV-2 infection as well as both the likelihood and severity of resulting illness [15]. We aimed to investigate the association between PCV13 receipt and risk of SARS-CoV-2 infection and related outcomes among recipients of COVID-19 vaccines.

## METHODS

### Setting

We undertook a retrospective study using data from the Kaiser Permanente Southern California (KPSC) healthcare system, which provides comprehensive, integrated healthcare to approximately 4.7 million members across virtual, outpatient, and inpatient settings. All diagnoses, laboratory test orders and results, prescriptions, procedures, and clinical notes are recorded in real time via patient electronic health records. Care received out-of-network is captured via insurance claims, and vaccination records are linked with the California Immunization Registry, enabling near-complete ascertainment of healthcare interactions for KPSC members.

### Design

We followed adults aged ≥65 years as of 1 January 2021 or 1 January 2022 through 31 December 2022 or exit from the cohort due to death or disenrollment. We restricted analyses to person-time from the first day after receipt of ≥2 COVID-19 vaccine doses, and to members enrolled in KPSC health plans since 1 January 2020 to ensure complete recording of comorbid conditions, vaccinations, and healthcare utilization throughout COVID-19 pandemic. We used a repeated-events survival analysis framework defining calendar months for each individual as the unit of analysis, thus accommodating changes in exposure status and time-varying confounders.

### Outcomes

The primary study outcome was confirmed SARS-CoV-2 infection, defined as a positive SARS-CoV-2 molecular test result in any clinical setting, without any record of SARS-CoV-2 infection within the preceding 60 days. We excluded infections ascertained via other means, including tests performed by other (non-KPSC) providers as well as self-test results uploaded to members’ online healthcare portals, as well as person-time accruing within 60 days following such events.

We additionally evaluated COVID-19–related hospital admission or death as secondary outcomes. We considered any inpatient admission occurring 7 days before to 28 days after a positive SARS-CoV-2 molecular test to be COVID-19 related; we further distinguished COVID-19–related inpatient admissions as being associated with acute respiratory illness (ARI) if individuals received any ARI diagnosis code during their hospital stay (Supplementary Table 1). We defined COVID-19–related deaths as those occurring up to 60 days after a positive SARS-CoV-2 test result. We right-censored observations each month for disenrollment, COVID-19–unrelated death (defined as any death without a positive SARS-CoV-2 test result within the preceding 60 days), or receipt of any vaccine that was accounted for within our statistical analysis, as detailed below (PCV13, any COVID-19 vaccine, 23-valent pneumococcal polysaccharide vaccine [PPSV23], zoster vaccine, or seasonal influenza vaccine). For analyses of ARI-associated hospital admissions related to COVID-19, we further censored at admissions without ARI diagnoses.

To minimize the likelihood for outcome misclassification due to unascertained SARS-CoV-2 infections, and to mitigate confounding from variation in testing behavior, our primary analyses included follow-up time when eligible KPSC members were actively engaged in SARS-CoV-2 testing, defined as periods during which members had received ≥1 SARS-CoV-2 test within the preceding 3 months. We repeated analyses restricting to periods during which members had received ≥1 SARS-CoV-2 test within the preceding 2 months or within the preceding month to ensure results were consistent with those obtained with more stringent selection on testing behavior. We also repeated analyses using a matched cohort framework as an alternative approach to mitigate bias. These analyses included all follow-up time from eligible KPSC members and matched members each month on the number of SARS-CoV-2 tests received in the preceding 1–3 months.

### Statistical Analysis

We estimated adjusted hazard ratios (aHRs) comparing risk for each outcome among recipients or nonrecipients of PCV13 using Cox proportional hazards models. Models matched on calendar time (defined via 6-month periods for January–June and July–December within each year), age, Advisory Committee on Immunization Practices (ACIP) risk stratum (Supplementary Table 2), the number of COVID-19 vaccine doses individuals had received and whether any doses were received within the preceding 6 months, history of prior recorded SARS-CoV-2 infections at any time before the follow-up interval, and prior receipt of PPSV23, zoster vaccine, and influenza vaccine for the current or most recent season. Analyses were further adjusted for individuals’ race or ethnicity, sex, and Charlson Comorbidity Index (CCI) score; strata for each covariate are defined in Table 1. We used the sandwich estimator to correct variance estimates for repeated observations of individuals.

**Table 1.** Characteristics of the Study Population

Characteristic	Full Study Population, by End of Enrollment, No. (%)^a^		Analytic Sample, by End of Enrollment, No. (%)^a,b^	
	PCV13 Unvaccinated (n *=* 112 284)	PCV13 Vaccinated (n *=* 484 801)	PCV13 Unvaccinated (n = 54 495)	PCV13 Vaccinated (n = 303 085)
Age, y				
65–69	82 115 (73.1)	108 581 (22.4)	41 363 (75.9)	69 207 (22.8)
70–74	16 077 (14.3)	150 123 (31.0)	6996 (12.8)	94 972 (31.3)
75–79	7828 (7.0)	105 684 (21.8)	3504 (6.4)	67 041 (22.1)
80–84	3717 (3.3)	64 374 (13.3)	1627 (3.0)	39 188 (12.9)
85–89	1684 (1.5)	36 283 (7.5)	709 (1.3)	21 505 (7.1)
≥90	863 (0.8)	19 756 (4.1)	296 (0.5)	11 172 (3.7)
Sex				
Male	50 512 (45.0)	217 561 (44.9)	23 728 (43.5)	138 183 (45.6)
Female	61 772 (55.0)	267 240 (55.1)	30 767 (56.5)	164 902 (54.4)
Race/ethnicity				
White, non-Hispanic	48 136 (42.9)	223 911 (46.2)	24 165 (44.3)	141 624 (46.7)
Black, non-Hispanic	12 837 (11.4)	43 848 (9.0)	6530 (12.0)	27 678 (9.1)
Hispanic ethnicity, any race	31 451 (28.0)	140 314 (28.9)	15 340 (28.1)	88 084 (29.1)
Asian	12 191 (10.9)	62 984 (13.0)	6059 (11.1)	38 367 (12.7)
Pacific Islander	589 (0.5)	3386 (0.7)	286 (0.5)	2028 (0.7)
Native American/Alaska Native	266 (0.2)	1112 (0.2)	130 (0.2)	654 (0.2)
Multiple	180 (0.2)	715 (0.1)	114 (0.2)	448 (0.1)
Other	1893 (1.7)	3946 (0.8)	784 (1.4)	2188 (0.7)
Unknown	4741 (4.2)	4585 (0.9)	1087 (2.0)	2014 (0.7)
ACIP risk stratum				
Low risk	76 322 (68.0)	167 348 (34.5)	34 271 (62.9)	92 053 (30.4)
At risk	29 771 (26.5)	221 782 (45.7)	16 564 (30.4)	144 217 (47.6)
High risk	6191 (5.5)	95 671 (19.7)	3660 (6.7)	66 815 (22.0)
CCI score				
0–1	87 313 (77.8)	194 918 (40.2)	39 759 (73.0)	109 060 (36.0)
2–3	17 439 (15.5)	151 425 (31.2)	10 031 (18.4)	95 911 (31.6)
4–5	4880 (4.3)	80 370 (16.6)	2871 (5.3)	53 895 (17.8)
≥6	2652 (2.4)	58 088 (12.0)	1834 (3.4)	44 219 (14.6)
Comorbid conditions				
Myocardial infarction	2330 (2.1)	32 193 (6.6)	1401 (2.6)	23 302 (7.7)
Congestive heart failure	2776 (2.5)	47 917 (9.9)	1720 (3.2)	35 879 (11.8)
Peripheral vascular disease	17 498 (15.6)	235 049 (48.5)	10 778 (19.8)	160 520 (53.0)
Cerebrovascular disease	3677 (3.3)	47 429 (9.8)	2209 (4.1)	33 326 (11.0)
Dementia	1336 (1.2)	25 246 (5.2)	671 (1.2)	15 271 (5.0)
COPD	11 233 (10.0)	113 027 (23.3)	7094 (13.0)	80 409 (26.5)
Rheumatic disease	1537 (1.4)	15 848 (3.3)	902 (1.7)	11 120 (3.7)
Ulcer	529 (0.5)	6745 (1.4)	386 (0.7)	5364 (1.8)
Paraplegia	718 (0.6)	7120 (1.5)	410 (0.8)	5064 (1.7)
Renal disease	8703 (7.8)	121 983 (25.2)	4938 (9.1)	81 253 (26.8)
Liver disease (mild)	4755 (4.2)	38 162 (7.9)	3013 (5.5)	27 765 (9.2)
Liver disease (moderate/severe)	219 (0.2)	2850 (0.6)	146 (0.3)	2377 (0.8)
Metastatic solid tumor	1001 (0.9)	11 483 (2.4)	731 (1.3)	8882 (2.9)
Carcinoma	4698 (4.2)	41 884 (8.6)	3056 (5.6)	30 575 (10.1)
HIV	14 (<0.1)	212 (<0.1)	10 (<0.1)	141 (<0.1)
Diabetes (uncomplicated)	11 541 (10.3)	95 446 (19.7)	5647 (10.4)	58 711 (19.4)
Diabetes (complicated)	7246 (6.5)	119 296 (24.6)	4031 (7.4)	80 191 (26.5)
Apnea	6398 (5.7)	56 231 (11.6)	4287 (7.9)	42 455 (14.0)
Depression	10 286 (9.2)	91 521 (18.9)	6281 (11.5)	63 128 (20.8)
Hypertension	47 769 (42.5)	347 875 (71.8)	25 585 (46.9)	221 916 (73.2)
Hypothyroid	10 259 (9.1)	78 430 (16.2)	5697 (10.5)	51 229 (16.9)
Asthma	8368 (7.5)	71 284 (14.7)	5395 (9.9)	51 567 (17.0)
SOT	41 (<0.1)	822 (0.2)	30 (0.1)	734 (0.2)
Other immunocompromise or immunodeficiency, not listed above	5096 (4.5)	47 279 (9.8)	3247 (6.0)	34 900 (11.5)
No. of COVID-19 vaccine doses received				
2	25 311 (22.5)	58 406 (12.0)	10 283 (18.9)	31 836 (10.5)
3	39 898 (35.5)	144 822 (29.9)	18 985 (34.8)	87 955 (29.0)
4	27 520 (24.5)	141 609 (29.2)	14 375 (26.4)	90 402 (29.8)
≥5	19 555 (17.4)	139 964 (28.9)	10 852 (19.9)	92 892 (30.6)
Other vaccines received				
PPSV23	64 865 (57.8)	471 549 (97.3)	35 459 (65.1)	296 788 (97.9)
Zoster vaccine	44 978 (40.1)	346 517 (71.5)	25 385 (46.6)	223 589 (73.8)
Influenza vaccine (any season)	75 910 (67.6)	464 809 (95.9)	40 305 (74.0)	293 376 (96.8)
No. of confirmed SARS-CoV-2 infections				
0	93 260 (83.1)	399 800 (82.5)	38 185 (70.1)	225 357 (74.4)
1	18 292 (16.3)	81 566 (16.8)	15 586 (28.6)	74 314 (24.5)
≥2	732 (0.7)	3435 (0.7)	724 (1.3)	3414 (1.1)

Abbreviations: ACIP, Advisory Committee on Immunization Practices; CCI, Charlson Comorbidity Index; COPD, chronic obstructive pulmonary disease; COVID-19, coronavirus disease 2019; HIV, human immunodeficiency virus; PCV13, 13-valent pneumococcal conjugate vaccine; PPSV23, 23-valent pneumococcal polysaccharide vaccine; SARS-CoV-2, severe acute respiratory syndrome coronavirus 2; SOT, solid organ transplantation.

^a^We define characteristics of individuals according to their last date of enrollment during the study period (ie, date of death, end of coverage, or 31 December 2022, whichever is earliest). For ACIP risk group, CCI score, COVID-19 vaccine doses received, and number of confirmed SARS-CoV-2 infections, values in the table correspond to the greatest value observed for each individual. When defining prevalence of each comorbid condition, we tabulate the number of individuals who were observed to have each condition at any point during their follow-up.

^b^The population eligible for inclusion is comprised of individuals who received at least 1 SARS-CoV-2 test during the study period.

As an additional effect measure, we estimated the absolute incidence of each outcome averted by PCV13. We obtained counterfactual incidence rates for PCV13 recipients, had they not received PCV13, by multiplying observed rates for each outcome among PCV13 recipients by the inverse of the aHR of the same outcome comparing PCV13 recipients to nonrecipients. We subtracted observed incidence rates from this counterfactual rate to obtain incidence averted.

To test the hypothesis that specific protection against SARS-CoV-2 would constrain the magnitude of PCV13 effects, we also assessed modification of PCV13 effectiveness associated with prior COVID-19 vaccination or SARS-CoV-2 infection. We repeated analyses within subgroups stratified by person-time following receipt of 2, 3, or ≥4 COVID-19 vaccine doses; person-time accruing within or beyond a 6-month time window from receipt of individuals’ most recent COVID-19 vaccine dose; and person-time accrued among individuals with or without ≥1 prior documented SARS-CoV-2 infection. We repeated these analyses limiting observations to person-time from 2022 only, after establishment of the Omicron variant, so that effects could not be driven by differences in circulating variants when comparing PCV13 effects among individuals who received 2, 3, and ≥4 vaccine doses.

Last, we estimated PCV13-associated protection against SARS-CoV-2 infection replicating a negative-control statistical framework employed in our earlier study [9, 16]. Under this approach, we included receipt of vaccines other than PCV13 as covariates for adjustment rather than using these to define matching strata. We divided estimates of the aHR comparing PCV13 recipients to nonrecipients by estimates of the aHR comparing zoster vaccine recipients to nonrecipients. Models again defined matching strata for COVID-19 vaccination history and calendar time and corrected for differences in the remaining adjustment variables via inverse propensity weighting (prior recorded SARS-CoV-2 infection, ACIP risk group, age group, race/ethnicity, sex, and CCI score). Zoster vaccination was considered a negative-control exposure due to a lack of known mechanisms by which zoster vaccination could affect individuals’ risk of COVID-19 outcomes, and the assumption that confounding pathways leading to any association between zoster vaccination and COVID-19 risk may be similar to those involving PCV13. However, we did not use this approach for primary analyses because implementation of shared clinical decision-making recommendations for PCV13 among most adults aged ≥65 years could lead to differences in the factors driving PCV13 uptake among adults becoming eligible for PCV13 during 2020–2022, and because other studies have interpreted associations between zoster vaccination and COVID-19 outcomes as evidence of vaccine-nonspecific effects [17].

## RESULTS

Our primary analyses included 357 580 unique individuals, among whom 303 085 (84.8%) received PCV13 at any point (Table 1). A greater share of individuals who remained nonrecipients of PCV13 throughout follow-up were aged 65–69 years, consistent with the change in 2019 from a recommendation for universal receipt of PCV13 among adults aged ≥65 years to use based on shared clinical decision-making. Aside from this difference, recipients generally had received greater numbers of COVID-19 vaccine doses; were more likely to have received other vaccines including PPSV23, zoster vaccines, and seasonal influenza vaccines; and had poorer health status, as reflected by CCI score distributions, frequencies of individual comorbid conditions, and the proportions belonging to the ACIP “at-risk” or “high-risk” strata for pneumococcal disease. In comparison to all eligible KPSC members aged ≥65 years during the study period (N = 597 085), those contributing person-time to primary analyses (based on receipt of SARS-CoV-2 testing) had higher prevalence of comorbid conditions, received greater numbers of COVID-19 vaccine doses and were more likely to receive other vaccines, and were more likely to have prior recorded SARS-CoV-2 infections at any point during analyses.

Incidence rates of confirmed SARS-CoV-2 infection varied over the study period (Supplementary Table 3). Throughout the study period, unadjusted rates of confirmed SARS-CoV-2 infection following receipt of ≥2 COVID-19 vaccine doses were 46.8 and 61.8 per 100 person-years (PY) among PCV13 recipients and nonrecipients, respectively. Adjusted hazard ratios for confirmed SARS-CoV-2 infection comparing PCV13 recipients to nonrecipients were 0.92 (95% confidence interval [CI], .90–.95) following receipt of ≥2 COVID-19 vaccine doses and 0.95 (95% CI, .92–.98) following receipt of ≥3 COVID-19 vaccine doses (Table 2). Corresponding estimates of the incidence of confirmed SARS-COV-2 infection averted by PCV13 were 3.9 (95% CI, 2.6–5.3) per 100 PY following receipt of ≥2 COVID-19 vaccine doses, and 2.9 (95% CI, 1.0–4.9) per 100 PY following receipt of ≥3 COVID-19 vaccine doses.

**Table 2.** Association of 13-Valent Pneumococcal Conjugate Vaccine Receipt With Risk of Confirmed Severe Acute Respiratory Syndrome Coronavirus 2 Infection

Vaccination or Infection History	Unadjusted Rate	Adjusted Effect Size Measures	
	Events per 100 PY at Risk (Total Cases Observed)	Hazard Ratio (95% CI)	Averted Cases per 100 PY (95% CI)
≥2 COVID-19 vaccine doses			
PCV13 not received	61.8 (13 879)	ref.	…
PCV13 received	46.8 (66 561)	0.92 (.90–.95)	3.9 (2.6–5.3)
≥3 COVID-19 vaccine doses			
PCV13 not received	73.9 (9673)	ref.	…
PCV13 received	60.0 (52 055)	0.95 (.92–.98)	2.9 (1.0–4.9)

Hazard ratios are computed via Cox proportional hazards regression models matching on COVID-19 vaccine doses received; calendar time (January–June or July–December for each of 2021 and 2022); receipt of pneumococcal polysaccharide vaccine, zoster vaccine, and same-season influenza vaccine; Advisory Committee on Immunization Practices–defined risk group (low risk, at risk, or high risk; Supplementary Table 2); cumulative confirmed severe acute respiratory syndrome coronavirus 2 (SARS-CoV-2) infections (0, 1, or ≥2); and age (65–69, 70–74, 75–79, 80–84, 85–89, or ≥90 years). Analyses further adjust for race/ethnicity (White, Black, Hispanic, Asian, Pacific Islander, Native American/Alaska Native, other, or unknown), sex, and Charlson Comorbidity Index score (0–1, 2–3, 4–5, or ≥6) via covariate adjustment. Eligible person-months are those preceded by a 3-month period in which individuals received at least 1 SARS-CoV-2 test; person-months at risk exclude those within ≤2 months after each documented SARS-CoV-2 infection, during which future positive test results would not be considered to represent new incident infections. We define observation units as calendar months for each individual, censoring at receipt of any vaccine, death, or disenrollment. We use the sandwich estimator to correct variance for repeated observations from individuals.

Abbreviations: CI, confidence interval; COVID-19, coronavirus disease 2019; PCV13, 13-valent pneumococcal conjugate vaccine; PY, person-years.

The aHR estimates closely resembled those of the primary analyses when considering alternative period lengths for matching of calendar time (Supplementary Table 4), when eligible person-time was restricted to periods when individuals had received ≥1 SARS-CoV-2 test within the preceding 2 months or the preceding month (Supplementary Table 5), and when matching on prior SARS-CoV-2 testing rather than restricting person-time contributions to periods following receipt of testing (Supplementary Table 6). In contrast, analyses that did not correct for differences in testing behavior by either restriction or matching yielded attenuated effect size estimates (aHRs estimated at 0.97 [95% CI, .94–.99] for recipients of ≥2 COVID-19 vaccine doses and 1.01 [95% CI, .98–1.04] for recipients of ≥3 COVID-19 vaccine doses). In confirmatory analyses using zoster vaccination as a negative-control exposure, aHR estimates for confirmed SARS-CoV-2 infection comparing PCV13 recipients to nonrecipients after ≥2 and ≥3 COVID-19 vaccine doses were 0.86 (95% CI, .82–.90) and 0.95 (95% CI, .89–1.01), respectively (Table 3).

**Table 3.** Sensitivity Analysis: Association of 13-Valent Pneumococcal Conjugate Vaccine Receipt With Risk of Confirmed Severe Acute Respiratory Syndrome Coronavirus 2 Infection, Based on Negative-Control Framework

Vaccination History	Adjusted Hazard Ratio (95% CI)
≥2 COVID-19 vaccine doses	
PCV13 not received	ref.
PCV13 received	0.86 (.82–.90)
≥3 COVID-19 vaccine doses	
PCV13 not received	ref.
PCV13 received	0.95 (.89–1.01)

Hazard ratios are computed via Cox proportional hazards regression models with negative-control correction as described in (ref.), dividing the adjusted hazard ratio (aHR) estimate for PCV13 by the aHR estimate for zoster vaccination. Models are fitted with stabilized inverse propensity weighting to balance distributions of risk factors among PCV13 recipients and nonrecipients. Covariates included in inverse propensity weighting include COVID-19 vaccine doses received; calendar time (January–June or July–December for each of 2020, 2021, and 2022); receipt of pneumococcal polysaccharide vaccine, zoster vaccine, and same-season influenza vaccine; Advisory Committee on Immunization Practices–defined risk group (low risk, at risk, or high risk); cumulative confirmed severe acute respiratory syndrome coronavirus 2 (SARS-CoV-2) infections (0, 1, or ≥2); age (65–69, 70–74, 75–79, 80–84, 85–89, or ≥90 years); race/ethnicity (White, Black, Hispanic, Asian, Pacific Islander, Native American/Alaska Native, other, or unknown); sex; and Charlson Comorbidity Index score (0–1, 2–3, 4–5, or ≥6). To account for test-seeking behavior, eligible person-months are those preceded by a 3-month period in which individuals received at least 1 SARS-CoV-2 test. We define observation units as calendar months for each individual, censoring at receipt of any vaccine, death, or disenrollment. Eligible person-months are those preceded by a 3-month period in which individuals received at least 1 SARS-CoV-2 test; person-months at risk exclude those within ≤2 months after each documented SARS-CoV-2 infection, during which future positive test results would not be considered to represent new incident infections. We use the sandwich estimator to correct variance for repeated observations from individuals.

Abbreviations: CI, confidence interval; COVID-19, coronavirus disease 2019; PCV13, 13-valent pneumococcal conjugate vaccine.

The aHR estimates for confirmed SARS-CoV-2 infection comparing PCV13 recipients to nonrecipients following receipt of 2, 3, and ≥4 COVID-19 vaccine doses were 0.85 (95% CI, .81–.89), 0.94 (95% CI, .90–.97), and 0.99 (95% CI, .93–1.04), respectively (Table 4). Analyses restricted to data from 2022 yielded similar estimates (Supplementary Table 7). Incidence averted by PCV13 amounted to 4.7 (95% CI, 3.3–6.3), 3.5 (95% CI, 1.5–5.6), and 1.1 (95% CI, −3.2 to 5.6) confirmed SARS-CoV-2 infections per 100 PY, respectively, following receipt of 2, 3, and ≥4 COVID-19 vaccine doses.

**Table 4.** Association of 13-Valent Pneumococcal Conjugate Vaccine Receipt With Risk of Confirmed Severe Acute Respiratory Syndrome Coronavirus 2 Infection, According to Number and Timing of Coronavirus Disease 2019 Vaccine Doses Received

Vaccination History	Unadjusted Rate	Adjusted Effect Size Measures	
	Events per 100 PY at Risk (Total Cases Observed)	Hazard Ratio (95% CI)	Averted Cases per 100 PY (95% CI)
2 COVID-19 vaccine doses			
PCV13 not received	44.9 (4206)	ref.	…
PCV13 received	26.2 (14 506)	0.85 (.81–.89)	4.7 (3.3–6.3)
3 COVID-19 vaccine doses			
PCV13 not received	65.1 (6450)	ref.	…
PCV13 received	50.7 (30 693)	0.94 (.90–.97)	3.5 (1.5–5.6)
≥4 COVID-19 vaccine doses			
PCV13 not received	101.3 (3223)	ref.	…
PCV13 received	81.2 (21 362)	0.99 (.93–1.04)	1.1 (–3.2 to 5.6)
≥2 COVID-19 vaccine doses, with no doses received within preceding 6 mo			
PCV13 received	88.0 (6375)	ref.	…
PCV13 not received	67.4 (26 007)	0.90 (.86–.93)	6.8 (4.3–9.2)
≥2 COVID-19 vaccine doses, with ≥1 dose received within preceding 6 mo			
PCV13 received	49.4 (7504)	ref.	…
PCV13 not received	39.1 (40 554)	0.94 (.91–.98)	2.3 (.9–3.5)

Hazard ratios are computed via Cox proportional hazards regression models matching on COVID-19 vaccine doses received (2, 3, 4, or ≥5); calendar time (January–June or July–December for each of 2020, 2021, and 2022); receipt of pneumococcal polysaccharide vaccine, zoster vaccine, and same-season influenza vaccine; Advisory Committee on Immunization Practices–defined risk group (low risk, at risk, or high risk); cumulative confirmed severe acute respiratory syndrome coronavirus 2 (SARS-CoV-2) infections (0, 1, or ≥2); and age (65–69, 70–74, 75–79, 80–84, 85–89, or ≥90 years). Analyses further adjust for race/ethnicity (White, Black, Hispanic, Asian, Pacific Islander, Native American/Alaska Native, other, or unknown), sex, and Charlson Comorbidity Index score (0–1, 2–3, 4–5, or ≥6). To account for test-seeking behavior, eligible person-months are those preceded by a 3-month period in which individuals received at least 1 SARS-CoV-2 test. We define observation units as calendar months for each individual, censoring at receipt of any vaccine, death, or disenrollment. The first 2 person-months after each confirmed SARS-CoV-2 infection are excluded to avoid double-counting of single infections. We use the sandwich estimator to correct variance for repeated observations from individuals.

Abbreviations: CI, confidence interval; COVID-19, coronavirus disease 2019; PCV13, 13-valent pneumococcal conjugate vaccine; PY, person-years.

Among recipients of ≥2 COVID-19 vaccine doses who had not received any COVID-19 vaccine dose within 6 months, the aHR estimate for confirmed SARS-CoV-2 infection comparing PCV13 recipients to nonrecipients was 0.90 (95% CI, .86–.93; Table 4). For individuals who had received ≥1 COVID-19 vaccine dose within the preceding 6 months, the corresponding aHR estimate was 0.94 (95% CI, .91–.98). Incidence of confirmed SARS-CoV-2 infection averted by PCV13 for recipients of ≥2 COVID-19 vaccine doses amounted to 6.8 (95% CI, 4.3–9.2) and 2.3 (95% CI, .9–3.5) per 100 PY within periods >6 months and ≤6 months after receipt of a COVID-19 vaccine dose, respectively.

Among recipients of ≥2 COVID-19 vaccine doses without documented history of SARS-CoV-2 infection, the aHR estimate for confirmed SARS-CoV-2 infection comparing PCV13 recipients to nonrecipients was 0.92 (95% CI, .89–.94; Table 5). Among individuals known to have experienced ≥1 SARS-CoV-2 infection previously, the corresponding aHR estimate was 1.00 (95% CI, .90–1.12). Incidence of confirmed SARS-CoV-2 infection averted by PCV13 was 4.2 (95% CI, 2.8–5.4) and 0.0 (95% CI, −2.5 to 2.1) per 100 PY at risk among individuals without prior documented SARS-CoV-2 infection and those with prior documented SARS-CoV-2 infection, respectively, among recipients of ≥2 COVID-19 vaccine doses. Considering recency of both prior vaccination and SARS-CoV-2 infections, the aHR comparing confirmed SARS-CoV-2 infection among PCV13 recipients versus nonrecipients was 0.89 (95% CI, .86–.93) among individuals who had not received any COVID-19 vaccine doses or experienced documented SARS-CoV-2 infection in the preceding 6 months, and 0.94 (95% CI, .91–.98) among individuals who either received COVID-19 vaccination or experienced documented SARS-CoV-2 infection over the same period (Supplementary Table 8).

**Table 5.** Association of 13-Valent Pneumococcal Conjugate Vaccine Receipt With Risk of Confirmed Severe Acute Respiratory Syndrome Coronavirus 2 (SARS-CoV-2) Infection Among Individuals With and Without Prior Documented SARS-CoV-2 Infection

Vaccination and Infection History	Receipt of PCV13	Unadjusted Rate	Adjusted Effect Size Measures	
		Events per 100 PY at Risk (Total Cases Observed)	Hazard Ratio (95% CI)	Averted Cases per 100 PY (95% CI)
≥2 COVID-19 vaccine doses				
No documented infection	PCV13 not received	68.2 (13 081)	ref.	…
	PCV13 received	50.4 (62 747)	0.92 (.89–.94)	4.2 (2.8–5.4)
≥1 documented infection	PCV13 not received	24.4 (798)	ref.	…
	PCV13 received	21.4 (3814)	1.00 (.90–1.12)	0.0 (–2.5 to 2.1)
≥3 COVID-19 vaccine doses				
No documented infection	PCV13 not received	82.9 (9140)	ref.	…
	PCV13 received	66.0 (49 162)	0.95 (.92–.98)	3.4 (1.2–5.3)
≥1 documented infection	PCV13 not received	25.7 (533)	ref.	…
	PCV13 received	23.5 (2893)	1.03 (.90–1.17)	–0.7 (–4.0 to 2.2)

Hazard ratios are computed via Cox proportional hazards regression models matching on COVID-19 vaccine doses received; calendar time (January–June or July–December for each of 2020, 2021, and 2022); receipt of pneumococcal polysaccharide vaccine, zoster vaccine, and same-season influenza vaccine; Advisory Committee on Immunization Practices–defined risk group (low risk, at risk, or high risk); cumulative confirmed severe acute respiratory syndrome coronavirus 2 (SARS-CoV-2) infections (0, 1, or ≥2); and age (65–69, 70–74, 75–79, 80–84, 85–89, or ≥90 years). Analyses further adjust for race/ethnicity (White, Black, Hispanic, Asian, Pacific Islander, Native American/Alaska Native, other, or unknown), sex, and Charlson Comorbidity Index score (0–1, 2–3, 4–5, or ≥6). To account for test-seeking behavior, eligible person-months are those preceded by a 3-month period in which individuals received at least 1 SARS-CoV-2 test. We define observation units as calendar months for each individual, censoring at receipt of any vaccine, death, or disenrollment. The first 2 person-months after each confirmed SARS-CoV-2 infection are excluded to avoid double-counting of single infections. We use the sandwich estimator to correct variance for repeated observations from individuals.

Abbreviations: CI, confidence interval; COVID-19, coronavirus disease 2019; PCV13, 13-valent pneumococcal conjugate vaccine; PY, person-years.

We did not identify evidence that PCV13 receipt was associated with differential risk of severe clinical outcomes of COVID-19 among recipients of ≥2 COVID-19 vaccine doses (Table 6). The aHR estimates comparing PCV13 recipients to nonrecipients were 1.00 (95% CI, .88–1.13) for any COVID-19–related hospital admission, and 1.10 (95% CI, .89–1.37) for COVID-19–related hospital admission with any ARI diagnosis recorded. For COVID-19–associated death, the aHR estimate comparing PCV13 recipients to nonrecipients was 1.15 (95% CI, .83–1.58).

**Table 6.** Association of 13-Valent Pneumococcal Conjugate Vaccine Receipt With Risk of Severe Clinical Outcomes of Coronavirus Disease 2019 (COVID-19) Among Recipients of ≥2 COVID-19 Vaccine Doses

Outcome and PCV13 Vaccination Status	Rate per 100 PY		Adjusted Effect Size Measures	
	Unadjusted Rate (Total Cases Observed)	Rate Standardized by Age, ACIP Risk Group, and Calendar Period	Hazard Ratio (95% CI)	Averted Cases per 100 PY (95% CI)
Any hospital admission				
PCV13 not received	1.44 (332)	3.14	ref.	…
PCV13 received	2.95 (4270)	2.94	0.99 (.86–1.14)	0.02 (–.43 to .40)
LRTI-related hospital admission				
PCV13 not received	0.59 (135)	1.31	ref.	…
PCV13 received	1.35 (1962)	1.35	1.10 (.89–1.37)	–0.14 (–.50 to .15)
Death				
PCV13 not received	0.10 (24)	0.37	ref.	…
PCV13 received	0.29 (414)	0.28	1.15 (.83–1.58)	–0.04 (–.16 to .05)

Hazard ratios are computed via Cox proportional hazards regression models matching on coronavirus disease 2019 vaccine doses received (2, 3, 4, or ≥5); calendar time (January–June or July–December for each of 2020, 2021, and 2022); receipt of pneumococcal polysaccharide vaccine, zoster vaccine, and same-season influenza vaccine; ACIP-defined risk group (low risk, at risk, or high risk); cumulative confirmed severe acute respiratory syndrome coronavirus 2 (SARS-CoV-2) infections (0, 1, or ≥2); and age (65–69, 70–74, 75–79, 80–84, 85–89, or ≥90 years). Analyses further adjust for race/ethnicity (White, Black, Hispanic, Asian, Pacific Islander, Native American/Alaska Native, other, or unknown), sex, and Charlson Comorbidity Index score (0–1, 2–3, 4–5, or ≥6). To account for test-seeking behavior, eligible person-months are those preceded by a 3-month period in which individuals received at least 1 SARS-CoV-2 test. We define observation units as calendar months for each individual, censoring at receipt of any vaccine, death, or disenrollment. We define observation units as calendar months for each individual, censoring at receipt of any vaccine, death, or disenrollment. The first 2 person-months after each confirmed SARS-CoV-2 infection are excluded to avoid double-counting of single infections. We use the sandwich estimator to correct variance for repeated observations from individuals.

Abbreviations: ACIP, Advisory Committee on Immunization Practices; CI, confidence interval; LRTI, lower respiratory tract infection; PCV13, 13-valent pneumococcal conjugate vaccine; PY, person-years.

## DISCUSSION

Among US older adults who received ≥2 COVID-19 vaccine doses, receipt of PCV13 was associated with modest clinical benefit amounting to an 8% reduction in incidence of confirmed SARS-CoV-2 infection, or prevention of roughly 4 confirmed SARS-CoV-2 infections per 100 PY at risk. This effect is weaker than that estimated within the same population prior to introduction of COVID-19 vaccines [9]. The magnitude of benefit associated with PCV13 receipt varied in association with variables known to predict the strength of individuals’ specific protection against outcomes related to SARS-CoV-2 infection. First, we identified incremental reductions in PCV13 effect sizes for prevention of confirmed SARS-CoV-2 infection among recipients of greater number of COVID-19 vaccine doses (15%, 6%, and 1% reductions in risk for recipients of 2, 3, and ≥4 COVID-19 vaccine doses, respectively). Second, PCV13 effect sizes were weaker for individuals who had received COVID-19 vaccine doses in a more recent timeframe (10% and 6% reduction in risk for individuals who had received their most recent COVID-19 vaccine dose >6 months or ≤6 months previously). Third, PCV13 effects were apparent for individuals without prior documented SARS-CoV-2 infection and inapparent for those with documented history of SARS-CoV-2 infection (8% and 0% reductions in risk, respectively). Last, we did not identify appreciable differences in risk of severe clinical outcomes against which COVID-19 vaccination is expected to provide robust protection (hospital admission, ARI-associated hospital admission, or deaths related to COVID-19) among PCV13 recipients and nonrecipients. These findings update understanding of the relationship between PCV13 receipt and clinical outcomes related to SARS-CoV-2 for the current context of broad COVID-19 vaccine uptake among US older adults.

Evidence from our study helps to define potential pathways of pneumococcal interaction with SARS-CoV-2. Studies undertaken during early phases of the COVID-19 pandemic, before COVID-19 vaccines became available, demonstrated higher pneumococcal colonization prevalence and density among adults with mild or asymptomatic SARS-CoV-2 infection than those without SARS-CoV-2 infection [3, 4]. Similarly, in a household-based longitudinal cohort study, pneumococcal colonization was associated with increased risk of acquisition of SARS-CoV-2 infection as well as increased duration of viral shedding [5]. In line with this finding, pneumococcal carriers experienced greater increases in risk of SARS-CoV-2 infection following exposure in comparison to corresponding increases in risk of SARS-CoV-2 infection following exposure among individuals who did not carry pneumococci [4]. Studies involving other viral infections have provided similar evidence that pneumococcal carriage modifies risk of ARI in a density-dependent manner [8] and modulates immune responses to live attenuated influenza vaccine [7].

Our results are consistent with a scenario in which PCV13, by reducing risk of vaccine-serotype pneumococcal carriage, reduces individuals’ risk of SARS-CoV-2 infection. However, studies are needed to confirm the role of this or other mechanisms in explaining these findings. While the effectiveness of PCV13 in preventing pneumococcal carriage among adults has been confirmed in challenge models [18], it is unclear if reduced carriage of these serotypes leads to a net reduction in pneumococcal carriage among adults, who likely encounter lower infectious pressure from nonvaccine serotypes in comparison to children [19, 20]. Serotype-specific pneumococcal interactions with influenza have been demonstrated [1] but remain uncertain for SARS-CoV-2 [21].

The interaction pathways discussed above are distinct from well-understood mechanisms of bacterial–viral co-pathogenesis whereby inflammation and epithelial injury resulting from primary viral infection exacerbate host susceptibility to subsequent bacterial infections. In our earlier study [9], PCV13 was associated with quantitatively similar reductions in adults’ risk of confirmed SARS-CoV-2 infection, COVID-19–related hospital admission, and COVID-19–related death. The lack of enhancement in protection against more-severe disease outcomes suggests that interactions with pneumococci do not necessarily exacerbate clinical progression of SARS-CoV-2, consistent with the low burden of secondary pneumococcal and other bacterial infections in COVID-19 patients [2]. These results have been replicated in subsequent analyses of healthcare claims data from US adults, showing that COVID-19 cases who had received PCV13 did not experience substantially lower risk of adverse clinical outcomes in comparison to COVID-19 cases who had not received PCV13 [22]. Very low incidence rates of COVID-19–related hospital admissions and deaths in our sample following receipt of ≥2 COVID-19 vaccine doses may have further contributed to the lack of appreciable differences in risk of these outcomes among PCV13 recipients and nonrecipients within the current study. This result is also consistent with our observations of attenuated PCV13 effect estimates in contexts where individuals’ history of COVID-19 vaccination or SARS-CoV-2 infection would be expected to afford stronger degrees of primary protection against the clinical outcomes analyzed. Regardless, it remains important to consider that our analyses of severe outcomes may encounter confounding if recipients of PCV13 are at greater risk for hospital admission or death due to factors beyond those included in our analysis. Nonspecific effects of PCV13 on cellular or mucosal responses to respiratory pathogens, although not widely studied, may also merit consideration in explaining our findings [23, 24].

Our study has several limitations. First, residual confounding due to unmeasured differences between PCV13 recipients and nonrecipients remains possible under our observational study design. Relatedly, our outcome of confirmed SARS-CoV-2 infection is imperfectly measured, as many infections may not have led to testing or diagnosis. Our findings hold within a sensitivity analysis aiming to correct for potential bias using inverse propensity weighting and defining zoster vaccination as a negative-control exposure [16], and in analyses that match (rather than restrict) eligible person-time based on SARS-CoV-2 testing behavior. Patterns of attenuation in PCV13 effect estimates are consistent with respect to the number of COVID-19 vaccine doses received, timing of most recent COVID-19 vaccination, and history of SARS-CoV-2 infection. Any individual source of confounding or bias would be unlikely to explain this consistency. Third, as our study was retrospective, we could not sample participants at baseline time points or at the point of SARS-CoV-2 testing to determine pneumococcal colonization status. Prospective studies employing such methodologies will offer a valuable strategy to understand mechanisms underpinning viral–bacterial interactions. Fourth, as third (and additional) COVID-19 vaccine doses were recommended at later stages of the study period, our comparisons of PCV13 effect sizes among recipients of 2, 3, and ≥4 COVID-19 vaccine doses may correspond to differing phases of the pandemic when differing variants were circulating. Last, data for this study precede the use of COVID-19 vaccines targeting BA.4/BA.5-related and XBB-related viral lineages as well as third-generation 15- and 20-valent PCVs. Continued monitoring of the incremental benefit of PCVs remains of interest with the recent introduction of these products.

In conclusion, our study suggests that PCV13 is associated with modest additional benefit for prevention of SARS-CoV-2 infection among adults who received COVID-19 vaccination. Highly effective COVID-19 vaccines now offer an important primary prevention strategy to mitigate the burden of SARS-CoV-2, meaning that benefits of PCV13 may be most pronounced for individuals with gaps in immunity against SARS-CoV-2. Such populations may include individuals who are not up-to-date with receipt of recommended numbers or types of COVID-19 vaccine doses, those with longer intervals since receipt of COVID-19 vaccine doses, or those who have not recently experienced SARS-CoV-2 infection [15, 25]. Determining the effectiveness of viral and bacterial vaccines in preventing differing clinical outcomes—including colonization as well illness involving targeted and coinfecting pathogens—may offer further insight into the pathogenesis and control of respiratory diseases. While past studies have demonstrated effects of PCVs in reducing severe virus-associated lower respiratory tract infections [10–13], knowledge of effects on lower-acuity endpoints remains incomplete. Studies uncovering pathways of viral–bacterial interaction remain an important priority to understand mechanisms by which PCVs may impact risk of virus-associated disease outcomes, including SARS-CoV-2 infection, and may inform integrated prevention approaches for respiratory illnesses.

## Supplementary Data

Supplementary materials are available at *The Journal of Infectious Diseases* online (http://jid.oxfordjournals.org/). Supplementary materials consist of data provided by the author that are published to benefit the reader. The posted materials are not copyedited. The contents of all supplementary data are the sole responsibility of the authors. Questions or messages regarding errors should be addressed to the author.

## Supplementary Material

jiae387_Supplementary_Data

## Notes

***Financial support*.** This study was sponsored by Kaiser Permanente Southern California with funding from Pfizer.
