# Supplementary material for: Association of Pneumococcal Conjugate Vaccination With Severe Acute Respiratory Syndrome Coronavirus 2 Infection Among Older Adult Recipients of Coronavirus Disease 2019 Vaccines: A Longitudinal Cohort Study
Source: J Infect Dis. 2024 Aug 5;230(5):e1082–91. doi: 10.1093/infdis/jiae387 (PMC11566223; doi:10.1093/infdis/jiae387)
Supplement: jiae387_Supplementary_Data [file jiae387_supplementary_data.pdf]

Supporting information

Association of pneumococcal conjugate vaccination with SARS-CoV-2 infection among older adult recipients of COVID-19 vaccines: a longitudinal cohort study

Joseph A. Lewnard[1,2,3,*], Vennis Hong[4], Lindsay R. Grant[5], Bradley K. Ackerson[4], Katia J. Bruxvoort[6], Magdalena Pomichowski[4], Adriano Arguedas[5], Alejandro Cané[5], Luis Jodar[5], Bradford D. Gessner[5], Sara Y. Tartof[4,7]

Affiliations:

1. School of Public Health, University of California, Berkeley, Berkeley, California 94720, United States Center for Computational Biology
2. College of Statistics, Data Science, and Society, University of California, Berkeley, Berkeley, California 94720, United States
3. Augmented Graduate Group in Computational Precision Health, University of California, Berkeley, Berkeley, California 94720, United States
4. Department of Research and Evaluation, Kaiser Permanente Southern California, Pasadena, California 91101, United States
5. Pfizer Vaccines, Collegeville, Pennsylvania 19426, United States
6. Department of Epidemiology, School of Public Health, University of Alabama at Birmingham, Birmingham, Alabama 35233, United States
7. Department of Health Systems Science, Kaiser Permanente Bernard J. Tyson School of Medicine, Pasadena, California 91101, United States

* Corresponding author:
Joseph A. Lewnard
Room 5410
2121 Berkeley Way
Berkeley, California 94720
Email: jLewnard@berkeley.edu
Tel.: 510-664-4050

Contents of this supplement

Item	Title	Page
Table S1	Acute respiratory infection diagnosis codes.	2
Table S2	Advisory Committee on Immunization Practices (ACIP)-defined risk strata for PCV13 receipt.	4
Table S3	Incidence of confirmed SARS-CoV-2 infection, by age, prior vaccination and calendar time.	5
Table S4	Association of PCV13 receipt with risk of confirmed SARS-CoV-2 infection among recipients of ≥2 COVID-19 vaccine doses, with matches defined over differing lengths of calendar time	6
Table S5	Association of PCV13 receipt with risk of confirmed SARS-CoV-2 infection within analyses restricting on test-seeking behavior.	7
Table S6	Association of PCV13 receipt with risk of confirmed SARS-CoV-2 infection within analyses matching on test-seeking behavior.	8
Table S7	Association of PCV13 receipt with risk of confirmed SARS-CoV-2 infection, according to number of COVID-19 vaccine doses received when restricting to follow-up during 2022	9
Table S8	Association of PCV13 receipt with risk of confirmed SARS-CoV-2 infection, according to timing of prior COVID-19 vaccination and documented SARS-CoV-2 infection	10

Table S1: Acute respiratory infection diagnosis codes.

ICD-10-CM Code	Diagnosis
A48.1	Legionnaire's disease
B34.2	Coronavirus infection (unspecified)
B44.0	Invasive pulmonary aspergillosis
B97.29	Other coronavirus as the cause of diseases classified elsewhere
J00	Acute nasopharyngitis (common cold)
J01.00	Acute maxillary sinusitis, unspecified
J01.10	Acute frontal sinusitis, unspecified
J01.20	Acute ethmoidal sinusitis, unspecified
J01.30	Acute sphenoidal sinusitis, unspecified
J01.40	Acute pansinusitis, unspecified
J01.80	Other acute sinusitis
J01.90	Acute sinusitis, unspecified
J02.0	Streptococcal pharyngitis
J02.8	Acute pharyngitis due to other specified organisms
J02.9	Acute pharyngitis, unspecified
J03.00	Acute streptococcal tonsillitis, unspecified
J03.90	Acute tonsillitis, unspecified
J04.0	Acute laryngitis
J04.10	Acute tracheitis without obstruction
J05.0	Acute obstructive laryngitis (croup)
J05.10	Acute epiglottitis without obstruction
J06.0	Acute laryngopharyngitis
J06.9	Acute upper respiratory infection, unspecified
J09.X1	Influenza due to identified novel influenza A virus with pneumonia
J09.X2	Influenza due to identified novel influenza A virus with other respiratory manifestations
J10.00	Influenza due to other identified influenza virus with unspecified type of pneumonia
J10.01	Influenza due to other identified influenza virus with same other identified influenza virus pneumonia
J10.08	Influenza due to other identified influenza virus with other pneumonia
J10.1	Influenza due to other identified influenza virus with other respiratory manifestations
J10.2	Influenza due to other identified influenza virus with gastrointestinal manifestations
J11.00	Influenza due to unidentified influenza virus with unspecified type of pneumonia
J11.08	Influenza due to unidentified influenza virus with specified pneumonia
J11.1	Influenza due to unidentified influenza virus with other respiratory manifestations
J12.1	Respiratory syncytial virus pneumonia
J12.2	Parainfluenza virus pneumonia
J12.3	Human metapneumovirus pneumonia
J12.81	Pneumonia due to SARS-associated coronavirus
J12.82	Pneumonia due to coronavirus disease 2019
J12.89	Other viral pneumonia
J12.9	Viral pneumonia, unspecified
J13	Pneumonia due to *Streptococcus pneumoniae*
J14	Pneumonia due to *Haemophilus influenzae*
J15.0	Pneumonia due to *Klebsiella pneumoniae*
J15.1	Pneumonia due to *Pseudomonas*
J15.20	Pneumonia due to *Staphylococcus*, unspecified
J15.211	Pneumonia due to methicillin susceptible *Staphylococcus aureus*
J15.212	Pneumonia due to methicillin resistant *Staphylococcus aureus*
J15.4	Pneumonia due to other *Streptococci*
J15.5	Pneumonia due to *Escherichia coli*
J15.6	Pneumonia due to other aerobic gram-negative bacteria
J15.7	Pneumonia due to *Mycoplasma pneumoniae*
J15.8	Pneumonia due to other specified bacteria
J15.9	Unspecified bacterial pneumonia
J16.8	Pneumonia due to other specified infectious organisms
J18.0	Bronchopneumonia, unspecified organism
J18.1	Lobar pneumonia, unspecified organism
J18.8	Other pneumonia, unspecified organism
J18.9	Pneumonia, unspecified organism
J20.2	Acute bronchitis due to *Streptococcus*
J20.5	Acute bronchitis due to respiratory syncytial virus
J20.6	Acute bronchitis due to rhinovirus
J20.8	Acute bronchitis due to other specified organisms
J20.9	Acute bronchitis, unspecified
J22	Unspecified acute lower respiratory infection
J39.0	Retropharyngeal and parapharyngeal abscess
J39.1	Other abscess of pharynx
J39.2	Other diseases of pharynx
J39.8	Other specified diseases of upper respiratory tract
J80	Acute respiratory distress syndrome
J96.00	Acute respiratory failure, unspecified with hypoxia or hypercapnia
J96.01	Acute respiratory failure with hypoxia
J96.02	Acute respiratory failure with hypercapnia
J96.10	Chronic respiratory failure, unspecified with hypoxia or hypercapnia
J96.11	Chronic respiratory failure with hypoxia

J96.12	Chronic respiratory failure with hypercapnia
J96.20	Acute and chronic respiratory failure, unspecified with hypoxia or hypercapnia
J96.21	Acute and chronic respiratory failure with hypoxia
J96.22	Acute and chronic respiratory failure with hypercapnia
J96.90	Respiratory failure, unspecified with hypoxia or hypercapnia
J96.91	Respiratory failure with hypoxia
J96.92	Respiratory failure with hypercapnia
M35.81	Multisystem inflammatory syndrome
M35.89	Other specified systemic involvement of connective tissue
R05.1	Acute cough
R05.3	Chronic cough
R05.8	Other specified cough
R05.9	Cough, unspecified
R09.2	Respiratory arrest
R50.9	Fever, unspecified
U07.1	COVID-19

Table S2: Advisory Committee on Immunization Practices (ACIP)-defined risk strata for PCV13 receipt.

High-risk (immunocompromised)	At-risk (Immunocompetent with comorbidity)
Presence of any of the following conditions:<br>• Congenital or acquired immunodeficiency<br>• Congenital or acquired asplenia<br>• Sickle cell disease/other hemoglobinopathies<br>• HIV infection<br>• Chronic renal failure<br>• Nephrotic syndrome<br>• Leukemia<br>• Lymphoma<br>• Hodgkin's disease<br>• Generalized malignancy<br>• Iatrogenic immunosuppression<br>• Solid organ transplantation<br>• Multiple myeloma<br>• Cerebrospinal fluid leak<br>• Cochlear implant	Presence of any of the following conditions, for individuals without immunosuppressed or immunocompromised status:<br>• Diabetes<br>• Chronic heart disease<br>• Chronic lung disease<br>• Chronic liver disease<br>• Alcoholism<br>• Smoking

Individuals not meeting criteria for "at risk" or "high risk" strata were considered to be "low risk".

Table S3: Incidence of confirmed SARS-CoV-2 infection, by age, prior vaccination and calendar time.

COVID-19 vaccination	Age group	PCV13 receipt	Unadjusted rate per 100 person-years (total cases observed)			
			January-June, 2021	*July-December, 2021*	*January-June, 2022*	*July-December, 2022*
2 COVID-19 vaccine doses						
	65-69 years					
		PCV13 not received	1.6 (22)	22.8 (593)	123.8 (1,479)	88.0 (606)
		PCV13 received	1.3 (27)	19.8 (779)	99.4 (699)	76.5 (319)
	70-74 years					
		PCV13 not received	0.9 (3)	21.6 (171)	105.6 (468)	79.2 (234)
		PCV13 received	1.2 (64)	19.0 (1,797)	104.8 (2,074)	75.9 (891)
	75-79 years					
		PCV13 not received	0.6 (1)	22.7 (91)	82.4 (168)	73.9 (101)
		PCV13 received	1.3 (57)	16.8 (1,259)	93.4 (1,479)	74.1 (716)
	80-84 years					
		PCV13 not received	– –	19.2 (33)	70.4 (67)	83.1 (53)
		PCV13 received	1.7 (46)	16.6 (719)	91.4 (912)	66.4 (419)
	85-89 years					
		PCV13 not received	– –	11.7 (9)	104.6 (44)	60.1 (19)
		PCV13 received	2.0 (29)	16.9 (398)	89.4 (559)	82.9 (336)
	≥90 years					
		PCV13 not received	– –	9.5 (3)	121.1 (27)	94.8 (14)
		PCV13 received	2.7 (21)	15.9 (202)	94.4 (435)	92.7 (269)
3 COVID-19 vaccine doses						
	65-69 years					
		PCV13 not received	– –	29.4 (196)	60.3 (2,623)	104.7 (1,871)
		PCV13 received	– –	20.2 (275)	54.0 (2,120)	92.7 (1,418)
	70-74 years					
		PCV13 not received	– –	20.2 (31)	55.1 (576)	95.1 (505)
		PCV13 received	– –	20.4 (669)	51.3 (6,233)	87.3 (3,768)
	75-79 years					
		PCV13 not received	– –	17.5 (13)	41.5 (192)	73.5 (185)
		PCV13 received	– –	17.3 (447)	44.0 (4,414)	80.5 (2,920)
	80-84 years					
		PCV13 not received	– –	6.6 (2)	28.8 (59)	70.7 (87)
		PCV13 received	– –	16.3 (227)	40.9 (2,428)	79.6 (1,779)
	85-89 years					
		PCV13 not received	– –	20.2 (3)	29.7 (25)	69.9 (35)
		PCV13 received	– –	11.1 (73)	40.4 (1,277)	84.8 (1,117)
	≥90 years					
		PCV13 not received	– –	22.3 (1)	50.8 (22)	83.2 (24)
		PCV13 received	– –	17.3 (58)	41.1 (733)	87.3 (737)
≥4 COVID-19 vaccine doses						
	65-69 years					
		PCV13 not received	– –	14.0 (1)	111.9 (421)	107.5 (2,038)
		PCV13 received	– –	10.2 (1)	88.3 (414)	92.8 (1,961)
	70-74 years					
		PCV13 not received	– –	– –	75.8 (57)	93.5 (379)
		PCV13 received	– –	26.2 (5)	78.7 (1,176)	89.3 (6,068)
	75-79 years					
		PCV13 not received	– –	– –	81.2 (29)	78.7 (156)
		PCV13 received	– –	– –	68.3 (932)	80.9 (4,916)
	80-84 years					
		PCV13 not received	– –	– –	79.4 (16)	68.6 (69)
		PCV13 received	– –	31.1 (2)	57.7 (468)	77.7 (2,829)
	85-89 years					
		PCV13 not received	– –	– –	65.1 (5)	107.5 (39)
		PCV13 received	– –	– –	55.7 (217)	74.8 (1,424)
	≥90 years					
		PCV13 not received	– –	– –	86.4 (2)	58.4 (11)
		PCV13 received	– –	– –	51.6 (104)	84.2 (845)

Eligible person-months are those preceded by a 3-month period in which individuals received at least one SARS-CoV-2 test; person-months at risk exclude those within ≤2 months after each documented SARS-CoV-2 infection, during which future positive test results would not be considered to represent new incident infections.

Table S4: Association of PCV13 receipt with risk of confirmed SARS-CoV-2 infection among recipients of ≥2 COVID-19 vaccine doses, with matches defined over differing lengths of calendar time.

Matched calendar time	Exposure	Adjusted hazard ratio (95% CI)
6 months (primary analysis)		
	PCV13 not received	ref.
	PCV13 received	0.92 (0.90, 0.95)
2 months		
	PCV13 not received	ref.
	PCV13 received	0.92 (0.90, 0.95)
3 months		
	PCV13 not received	ref.
	PCV13 received	0.93 (0.90, 0.95)
12 months		
	PCV13 not received	ref.
	PCV13 received	0.93 (0.90, 0.95)

Hazard ratios are computed via Cox proportional hazards regression models matching on: COVID-19 vaccine doses received; calendar time (January-June or July-December for each of 2021 and 2022); receipt of pneumococcal polysaccharide vaccine, zoster vaccine, and same-season influenza vaccine; ACIP-defined risk group (low risk, at risk, or high risk; **Table S2**); cumulative confirmed SARS-CoV-2 infections (0, 1, or ≥2); and age (defined as 5-year bins through ages 65-89, and as ≥90 years or greater). Analyses further adjust for race/ethnicity (White, Black, Hispanic, Asian, Pacific Islander, Native American/Alaska Native, Other, or Unknown), sex, and Charlson comorbidity index (binned as 0-1, 2-3, 4-5, or ≥6) via covariate adjustment. Eligible person-months are those preceded by a 3-month period in which individuals received at least one SARS-CoV-2 test; person-months at risk exclude those within ≤2 months after each documented SARS-CoV-2 infection, during which future positive test results would not be considered to represent new incident infections. We define observation units as calendar months for each individual, censoring at receipt of any vaccine, death, or disenrollment. We use the sandwich estimator to correct variance for repeated observations from individuals.

Table S5: Association of PCV13 receipt with risk of confirmed SARS-CoV-2 infection within analyses restricting on test-seeking behavior.

Adjustment	Adjusted hazard ratio (95% confidence interval)	
	≥2 COVID-19 vaccine doses	≥3 COVID-19 vaccine doses
Restriction to individuals tested within preceding 2 months	0.92 (0.89, 0.94)	0.95 (0.92, 0.98)
Restriction to individuals tested within preceding month	0.92 (0.90, 0.95)	0.95 (0.92, 0.94)

Hazard ratios are computed via Cox proportional hazards regression models matching on: COVID-19 vaccine doses received (2, 3, 4, or ≥5); calendar time (January-June or July-December for each of 2020, 2021, and 2022); receipt of pneumococcal polysaccharide vaccine, zoster vaccine, and same-season influenza vaccine; ACIP-defined risk group (low risk, at risk, or high risk); cumulative confirmed SARS-CoV-2 infections (0, 1, or ≥2); and age (defined as 5-year bins through ages 65-89, and as ≥90 years or greater). Analyses further adjust for race/ethnicity (White, Black, Hispanic, Asian, Pacific Islander, Native American/Alaska Native, Other, or Unknown), sex, and Charlson comorbidity index (binned as 0-1, 2-3, 4-5, or ≥6). We define observation units as calendar months for each individual, censoring at receipt of any vaccine, death, or disenrollment. Eligible person-months are those preceded by a 3-month period in which individuals received at least one SARS-CoV-2 test; person-months at risk exclude those within ≤2 months after each documented SARS-CoV-2 infection, during which future positive test results would not be considered to represent new incident infections. We use the sandwich estimator to correct variance for repeated observations from individuals.

Table S6: Association of PCV13 receipt with risk of confirmed SARS-CoV-2 infection within analyses matching on test-seeking behavior.

Adjustment	Adjusted hazard ratio (95% confidence interval)	
	≥2 COVID-19 vaccine doses	≥3 COVID-19 vaccine doses
Matching on number of tests received in preceding 3 months	0.92 (0.90, 0.95)	0.95 (0.92, 0.98)
Matching on receipt of any test in preceding 3 months	0.92 (0.90, 0.95)	0.95 (0.92, 0.98)
Matching on number of tests received in preceding 2 months	0.92 (0.89, 0.94)	0.95 (0.92, 0.98)
Matching on receipt of any test in preceding 2 months	0.92 (0.90, 0.95)	0.95 (0.92, 0.98)
Matching on number of tests received in preceding month	0.92 (0.89, 0.94)	0.95 (0.92, 0.98)
Matching on receipt of any test in preceding month	0.92 (0.89, 0.94)	0.95 (0.92, 0.98)

Hazard ratios are computed via Cox proportional hazards regression models matching on: for COVID-19 vaccine doses received (2, 3, 4, or ≥5); calendar time (January-June or July-December for each of 2020, 2021, and 2022); receipt of pneumococcal polysaccharide vaccine, zoster vaccine, and same-season influenza vaccine; ACIP-defined risk group (low risk, at risk, or high risk); cumulative confirmed SARS-CoV-2 infections (0, 1, or ≥2); and age (defined as 5-year bins through ages 65-89, and as ≥90 years or greater). Analyses further adjust for race/ethnicity (White, Black, Hispanic, Asian, Pacific Islander, Native American/Alaska Native, Other, or Unknown), sex, and Charlson comorbidity index (binned as 0-1, 2-3, 4-5, or ≥6). We define observation units as calendar months for each individual, censoring at receipt of any vaccine, death, or disenrollment. Eligible person-months are those preceded by a 3-month period in which individuals received at least one SARS-CoV-2 test; person-months at risk exclude those within ≤2 months after each documented SARS-CoV-2 infection, during which future positive test results would not be considered to represent new incident infections. We use the sandwich estimator to correct variance for repeated observations from individuals.

Table S7: Association of PCV13 receipt with risk of confirmed SARS-CoV-2 infection, according to number of COVID-19 vaccine doses received when restricting to follow-up during 2022.

Vaccination history		Adjusted hazard ratio (95% CI)
2 COVID-19 vaccine doses		
	PCV13 not received	ref.
	PCV13 received	0.83 (0.79, 0.88)
3 COVID-19 vaccine doses		
	PCV13 not received	ref.
	PCV13 received	0.95 (0.91, 0.98)
≥4 COVID-19 vaccine doses		
	PCV13 not received	ref.
	PCV13 received	1.00 (0.95, 1.06)

Hazard ratios are computed via Cox proportional hazards regression models matching on: COVID-19 vaccine doses received (2, 3, 4, or ≥5); calendar time (January-June or July-December of 2022); receipt of pneumococcal polysaccharide vaccine, zoster vaccine, and same-season influenza vaccine; ACIP-defined risk group (low risk, at risk, or high risk); cumulative confirmed SARS-CoV-2 infections (0, 1, or ≥2); and age (defined as 5-year bins through ages 65-89, and as ≥90 years or greater). Analyses further adjust for race/ethnicity (White, Black, Hispanic, Asian, Pacific Islander, Native American/Alaska Native, Other, or Unknown), sex, and Charlson comorbidity index (binned as 0-1, 2-3, 4-5, or ≥6). To account for test-seeking behavior, eligible person-months are those preceded by a 3-month period in which individuals received at least one SARS-CoV-2 test. We define observation units as calendar months for each individual, censoring at receipt of any vaccine, death, or disenrollment. The first two person-months after each confirmed SARS-CoV-2 infection are excluded to avoid double-counting of single infections. We use the sandwich estimator to correct variance for repeated observations from individuals.

Table S8: Association of PCV13 receipt with risk of confirmed SARS-CoV-2 infection, according to timing of prior COVID-19 vaccination and documented SARS-CoV-2 infection.

Vaccination and infection history		Unadjusted rate	Adjusted effect size measures	
		Events per 100 person-years at risk (total cases observed)	*Hazard ratio (95% CI)*	*Averted cases per 100 person-years (95% CI)*
≥2 COVID-19 vaccine doses, with no doses received and no documented SARS-CoV-2 infection within preceding 6 months				
	PCV13 received	96.8 (6,336)	ref.	– –
	PCV13 not received	72.3 (25,778)	0.89 (0.86, 0.93)	7.8 (5.1-10.4)
≥2 COVID-19 vaccine doses, with ≥1 dose received or any documented SARS-CoV-2 infection within preceding 6 months				
	PCV13 received	47.4 (7,543)	ref.	– –
	PCV13 not received	38.3 (40,783)	0.94 (0.91, 0.98)	2.1 (0.8-3.4)

Hazard ratios are computed via Cox proportional hazards regression models matching on: COVID-19 vaccine doses received (2, 3, 4, or ≥5); calendar time (January-June or July-December for each of 2020, 2021, and 2022); receipt of pneumococcal polysaccharide vaccine, zoster vaccine, and same-season influenza vaccine; ACIP-defined risk group (low risk, at risk, or high risk); cumulative confirmed SARS-CoV-2 infections (0, 1, or ≥2); and age (defined as 5-year bins through ages 65-89, and as ≥90 years or greater). Analyses further adjust for race/ethnicity (White, Black, Hispanic, Asian, Pacific Islander, Native American/Alaska Native, Other, or Unknown), sex, and Charlson comorbidity index (binned as 0-1, 2-3, 4-5, or ≥6). To account for test-seeking behavior, eligible person-months are those preceded by a 3-month period in which individuals received at least one SARS-CoV-2 test. We define observation units as calendar months for each individual, censoring at receipt of any vaccine, death, or disenrollment. The first two person-months after each confirmed SARS-CoV-2 infection are excluded to avoid double-counting of single infections. We use the sandwich estimator to correct variance for repeated observations from individuals.
